# IoT-Enabled Electronic Nose System for Beef Quality Monitoring and Spoilage Detection

**DOI:** 10.3390/foods12112227

**Published:** 2023-05-31

**Authors:** Asrar Nabil Damdam, Levent Osman Ozay, Cagri Kaan Ozcan, Ashwaq Alzahrani, Raghad Helabi, Kahled Nabil Salama

**Affiliations:** 1 Sensors Lab, Advanced Membranes and Porous Materials Center, Computer, Electrical and Mathematical Science and Engineering Division, King Abdullah University of Science and Technology (KAUST), Thuwal 23955-6900, Saudi Arabia; asrar.damdam@kaust.edu.sa; 2 Uvera Lab, Research and Development Department, Uvera Inc., Thuwal 23955-6900, Saudi Arabia

**Keywords:** IoT, food quality monitoring, beef quality, e-nose, food spoilage, food waste

## Abstract

Food spoilage is a major concern in the food industry, especially for highly perishable foods such as beef. In this paper, we present a versatile Internet of Things (IoT)-enabled electronic nose system to monitor food quality by evaluating the concentrations of volatile organic compounds (VOCs). The IoT system consists mainly of an electronic nose, temperature/humidity sensors, and an ESP32-S3 microcontroller to send the sensors’ data to the server. The electronic nose consists of a carbon dioxide gas sensor, an ammonia gas sensor, and an ethylene gas sensor. This paper’s primary focus is to use the system for identifying beef spoilage. Hence, the system performance was examined on four beef samples stored at different temperatures: two at 4 °C and two at 21 °C. Microbial population quantifications of aerobic bacteria, Lactic Acid Bacteria (LAB), and *Pseudomonas* spp., in addition to pH measurements, were conducted to evaluate the beef quality during a period of 7 days to identify the VOCs concentrations that are associated with raw beef spoilage. The spoilage concentrations that were identified using the carbon dioxide, ammonia, and ethylene sensors were 552 ppm–4751 ppm, 6 ppm–8 ppm, and 18.4 ppm–21.1 ppm, respectively, as determined using a 500 mL gas sensing chamber. Statistical analysis was conducted to correlate the bacterial growth with the VOCs production, where it was found that aerobic bacteria and *Pseudomonas* spp. are responsible for most of the VOCs production in raw beef.

## 1. Introduction

The issue of food loss and waste (FLW) has been recognized as a serious problem for food security, the environment, and economies. It has been estimated that about 1.3 billion tons of all the food produced for human consumption are lost or wasted throughout the food supply chain annually [1]. Moreover, the growing demand for meat is putting a major strain on the environment and the food supply chain’s overall sustainability as the meat industry is considered among the leading producers of greenhouse gases. The environmental cost of raising a cow for beef production is considerable—from water use and waste discharge down to greenhouse gas emissions due to intensive land use [2,3]. The total global meat production is currently estimated to be around 340 million tons; however, 23% of this amount is wasted throughout the entire supply chain [4]. These losses are mainly due to overproduction, poor handling and packaging methods, inappropriate storage and transportation conditions, inefficiencies during food processing, lack of infrastructure (such as refrigeration), lack of technical knowledge and skills, or by consumers [5,6]. Spoilage during storage and transportation is a major cause of meat loss across the supply chain. Although meat storage temperatures should not exceed 5 °C, the temperature increases during storage and transportation are thought to be a key factor in meat quality degradation, particularly during the summer. For instance, studies have revealed that the acceptable maximum temperature for refrigerated storage is frequently not fulfilled and that temperatures above 10 °C are not uncommon during transportation [7,8]. Meat can be damaged even before it reaches retail outlets, which is a widespread food safety risk existing across the meat supply chain. Hence, it is necessary to continuously monitor the quality of fresh meat during cold storage and transportation to ensure product freshness and safety, improve these processes, and reduce the loss and waste across the supply chain [9]. Several methods have been used to examine the food quality, such as microbial load counts, spectroscopic techniques [10,11,12,13], laser speckle imaging [11,14,15,16], E-tongues [17,18,19,20], and enzyme biosensors [10,21,22]. These methods have been able to successfully quantify and detect the freshness, shelf life, and early spoilage of various foods, i.e., fish, meat, and beverages. However, some of these methods are sample destructive, laborious, bulky, time-consuming, and require special training, while others are expensive, require frequent calibration, or cannot offer real-time monitoring [17,21,23]. In contrast, gas sensing is a quick, accurate, and operation-friendly method for detecting spoilage and food quality [9,10,21,24]. Every food product produces different aromas or gases consisting of a distinctive composition of volatile organic compounds (VOCs), which can indicate food quality, safety, and spoilage [25]. Moreover, owing to the effectiveness of gas sensing systems in providing real-time, simple, and cost-effective food quality monitoring, its applications are widespread in fruit, vegetable, seafood, beverage, and meat industries, and various researchers have studied the application of gas-sensing methods in food spoilage and ripening monitoring [10,11,23,24,25]. Among them, electronic nose systems (e-nose) have been used widely to monitor food ripening and detect spoilage. For instance, Wang et al. [26] developed an e-nose for the real-time freshness and spoilage evaluation of pork meat, banana, and leeks by analyzing hydrogen sulfide, ammonia, and ethylene production. The results showed that this method is effective in monitoring the freshness of sampled food in the refrigerator with a high accuracy that reaches 92% [26]. Similarly, [27] developed an e-nose using a six-sensor array with MOS sensors to monitor the levels of ethylene, alcohol, ammonia, and hydrogen produced from bananas. It was concluded that the sensor array’s capability for detecting ripening fingerprints is acceptable [27]. Rivai et al. [28] combined the usage of gas sensors and a neural network to study the ripeness state of durian to detect ethylene, hydrogen sulfide, and ethanol; this system successfully distinguished the ripeness of durian with an accuracy of about 91%. In another comprehensive study, Rivai et al. [29] evaluated the freshness of meat using a gas sensor array that detects H_2_S and NH_3_ with a neural network pattern recognition to accurately identify fresh and non-fresh meat samples [29]. A variety of gases can indicate the food quality, including alcohols, amines, and esters as well as organic compounds [24]. Hence, the selection of sensors for the electronic nose systems varies between studies based on the gases produced by the targeted food types. Carbon dioxide is among the primary indicators of food safety and freshness as it is a by-product of bacterial metabolism in the fresh produce ripening process [24,30]. Similarly, ammonia production is an important biomarker for the spoilage of various foods. In particular, the spoilage of protein-rich food (such as meat, pork, and fish) results in the production of nitrogenous compounds, including ammonia, dimethylamine, and trimethylamine, which are produced due to the decomposition of amino acids and microbiological activities [31,32]. Likewise, in post-harvest quality control and preservation, the role of ethylene gas is important as it affects the ripening of many fruits and vegetables [33]. To assess the fruit quality and delay spoilage, controlling the ethylene levels in the fruit distribution systems to regulate the ripening process is important [24]. Even in different storage conditions, such as modified atmospheres, the ethylene concentration should be carefully monitored as a low concentration is sufficient to trigger ripening in various fruits (i.e., climacteric fruits) [34]. Therefore, detecting ethylene is necessary to determine the ripeness or spoilage of fruits and vegetables. Generally, complex laboratory equipment (such as GC-MS) is used to detect the presence of ethylene; thus, the procedures for determining its quantity could be difficult and expensive [24]. There have been many efforts to develop universal, low-cost, and easy-to-use electronic nose systems for food quality monitoring and spoilage detection. Therefore, we present an IoT-enabled e-nose system for monitoring the overall food quality and detecting the spoilage of meats and fresh produce. Three gas sensors were selected for the e-nose system: an optical carbon dioxide gas sensor, a chemo-resistive ammonia gas sensor, and a chemo-resistive ethylene gas sensor. In this paper, we measure the gases produced from raw beef using the e-nose system and correlate the results with the conventional microbiological plate count to identify the spoilage and its gaseous concentrations. Since microbial growth, lipid oxidation, and enzymatic reactions are the main causes of meat deterioration [35], this paper also investigates the effect of aerobic bacteria, *Pseudomonas* spp., and Lactic Acid Bacteria (LAB) on the production of carbon dioxide, ammonia, and ethylene in raw beef using linear regression.

## 2. Materials and Methods

To identify the gas concentration levels that correspond to quality deterioration:

- The e-nose system measures the emissions of volatile organic compounds (VOCs) using the gas sensors array every 6 h.
- The beef quality is assessed daily by counting the microbial populations of Pseudomonas spp., Lactic Acid Bacteria (LAB), and aerobic bacteria, and performing Salmonella detection and pH levels measurements.
- Two shelf-life experimental runs were carried out for each storage temperature (4 °C and 21 °C) to verify the microbiological quantification results and validate the precision of the shelf-life estimation and gas concentration measurements.

### 2.1. Sample Preparation

Four fresh CAB boneless rib beef steaks were purchased from a local supermarket (Thuwal, Saudi Arabia) in January 2023. The samples’ weights ranged between 120 and 150 g. Within 15 min of being purchased, the samples were transported to the lab while being kept in thermal bags. The beef samples were stored separately in 1.4 L Eastman Tritan PCTG TX1001 plastic containers (STATUS, Metlika, Slovenia) and were split into two groups: the first group was stored in a fridge at 4 °C and the other one was stored at room temperature (21 °C).

### 2.2. Quality Monitoring System

#### 2.2.1. The Architecture Design of the IoT Monitoring System

**Figure 1:**
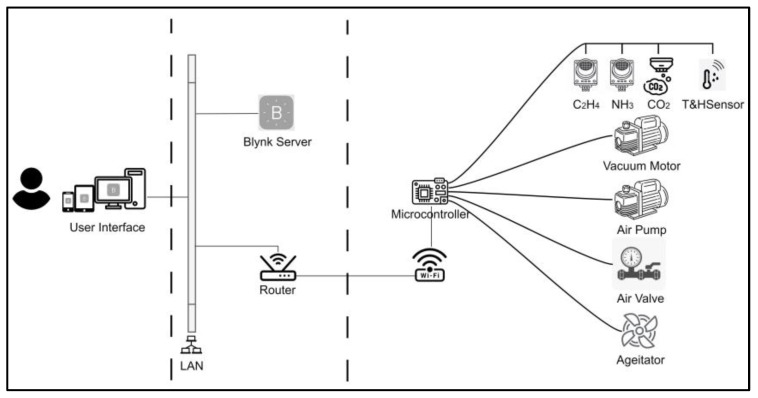
The IoT system architecture of the e-nose monitoring system.

The proposed IoT system consists of three main layers: (1) data sensing layer, (2) data transmission layer, and (3) user interface layer as shown in Figure 1. The data sensing layer features temperature, humidity, carbon dioxide, ammonia, and ethylene sensors. The data transmission layer enables remote data access via the Blynk server (Blynk Inc., New York, NY, USA), which uses HTTPs protocol to send the data from the sensing layer to the API. The user interface layer provides real-time monitoring with visual widgets in the Blynk system.

#### 2.2.2. The E-Nose System Architecture and Components

**Figure 2:**
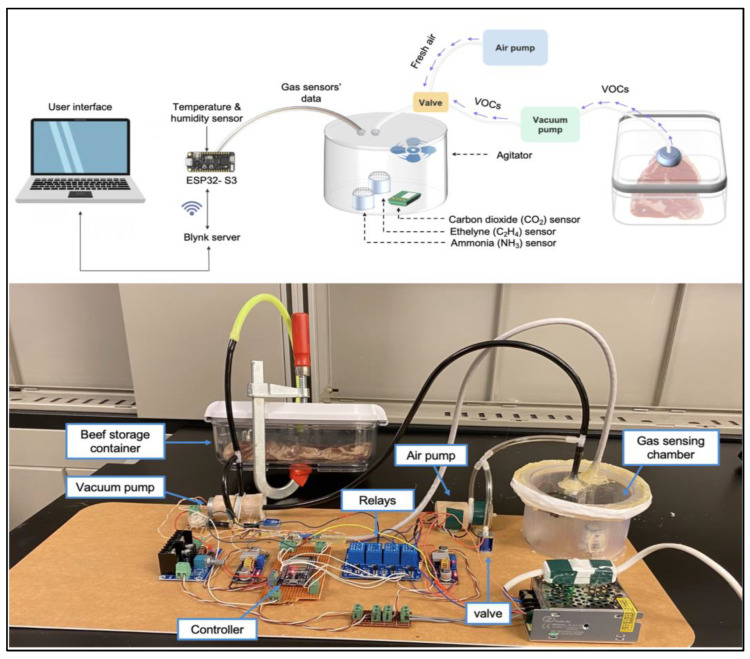
E-nose system.

The e-nose system consists of a cylindrical gas chamber that contains the gas sensor array and an agitator, temperature, and humidity sensors, a vacuum pump, an air pump, a valve, and a controller as illustrated in Figure 2. The gas chamber has a 500 mL volume, a 6.3 cm height, and a 5 cm base radius. The chamber body was 3D printed using a Form 3L printer (Formlabs Inc., Somerville, MA, USA) and SLA resin, while the lid was created by cutting a 0.6 cm thick acrylic sheet using Epilog 75 w laser cutter (Epilog Laser, Golden, CO, USA). A 0.5 cm thick silicone gasket was created using Sylgard 184 PDMS (Dow Corning, Freeland, MI, USA) to ensure the gas confinement inside the chamber. The sensors used in the e-nose system include an AM2302 temperature and humidity sensor (Aosong Electronics Co., Guangzhou, China), MH-Z19C carbon dioxide sensor, ZE03-NH3 ammonia sensor, and ZE03-C2H4 ethylene sensor (Winsen Electronics Technology Co., Zhengzhou, China). The detection range, response time, accuracy, and operating temperatures of the sensors are shown in Table 1. The main processing unit of the presented IoT system is the ESP32-S3 controller (Espressif Systems Shanghai Co., Shanghai, China), which has an extensive set of peripherals and can be easily connected to the internet using a native WiFi system. The ZE03-C2H4 and ZE03-NH3 sensors were connected to the ESP32-S3’s ADC pins, while the MH-Z19C gas sensor is connected to the UART1 channel. The AM2302 temperature sensor and relays were connected to the ESP32-S3’s digital I/O pins. The SC2701XPV vacuum and air pumps (Shenzhen Skoocom Electronic Co., Shenzhen, China) and GD3010-N agitator (Gulf Electrics Co., Kaohsiung City, Taiwan) were connected to the relays. The ESP32-S3 controller was programmed using the ESP-IDF framework. The code was written in C++ and included various algorithms for collecting and processing the sensor data, controlling the pumps and the valve (SC0526GF, Shenzhen Skoocom Electronic Co., Shenzhen, China), and sending the data to the cloud.

#### 2.2.3. The E-Nose Block Diagram

**Figure 3:**
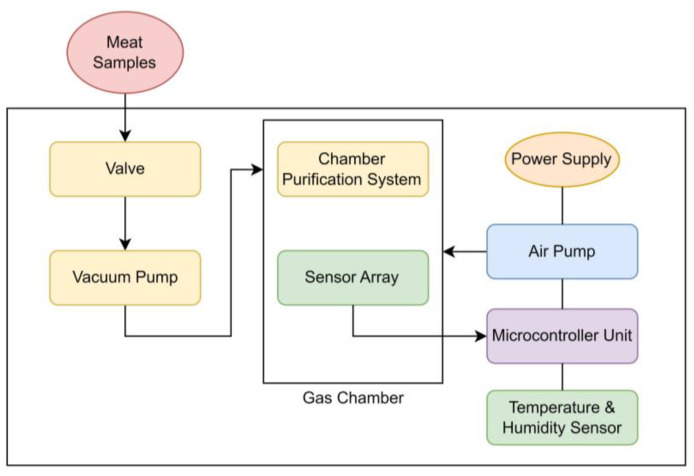
The block diagram of the electronic nose system.

Figure 3 shows the block diagram of the electronic nose system. The vacuum pump was used to suck the air from the beef containers and pump it into the gas chamber every 6 h. Various studies have demonstrated that the levels of VOCs produced by a variety of microorganisms start to increase after six hours [36,37]. Therefore, the 6-h time interval was chosen because the accumulation of VOCs in the storage medium is necessary for accurate spoilage detection. The e-nose system collects the data automatically for 15 min in every measurement cycle. The agitator ensures the gas composition’s homogeneity inside the e-nose chamber. After 12 min of reading the data, the air pump injects fresh air into the gas chamber. The valve controls the airflow between the container and the gas chamber.

### 2.3. Shelf Life and Quality Assessment

#### 2.3.1. Microbial Population Quantification

According to ISO standards, the aerobic mesophilic bacteria (ISO 4833-2:2013), *Pseudomonas* spp. (ISO 13720:2010), and lactic acid bacteria (ISO 13721:1995) were counted in both beef samples by using Plate Count Agar (PCA, Oxoid, Thermo Scientific, Basingstoke, UK), Pseudomonas CFC Agar Base (Oxoid, Thermo Scientific, Basingstoke, UK), SR0103E Pseudomonas C-F-C supplement and glycerol, and De Man, Rogosa Sharpe agar (MRS, Oxoid, Thermo Scientific, Basingstoke, UK), respectively. Salmonella sp. was also identified (ISO 6579:2002) using Rappaport Vassiliadis soya peptone broth (RVS, Condalab, Madrid, Spain), Muller–Kauffmann Tetrathionate Novobiocin Broth (MKTTn, Condalab, Madrid, Spain), and Xylose Lysine Deoxycholate (XLD, Condalab, Madrid, Spain). The instruments and workspace were sterilized with 70% alcohol. Five grams of each sample was then placed in stomacher bags and homogenized for 2 min in the stomacher with 45 mL of sterile physiological solution to produce the first suspension (10^−1^ dilution). For the second dilution (10^−2^), 1 mL of the first dilution was added to 9 mL of the physiological solution using a sterile pipette. More dilutions were created by repeating the same procedures. Then, 100 µL of each dilution was spread–plated into CFC and incubated at 25 °C for 48 h to determine the *Pseudomonas* spp. population, while 1 mL of each dilution was poured–plated into PCA and MRS and incubated at 30 °C for 72 h to determine the aerobic mesophilic bacteria and LAB populations. Salmonella sp. was detected by incubating the initial dilution for no more than 20 h, followed by enrichment of 1 mL in MKTTn broth and incubation at 37 °C for 24 h. Additionally, 0.1 mL of RVS broth was enriched and incubated for 24 h at 41.5 °C. After streaking 10 µL of each bouillon onto XLD Petri dishes, the dishes were incubated at 37 °C for 24 h. Daily microbial analyses were performed throughout the shelf life of each sample to evaluate the microbial growth, which influences the sensory properties and is used to determine the expiration of the beef samples. The colony-forming units per gram (CFU/g) were used to evaluate the results.

#### 2.3.2. pH Measurement

The pH levels were measured using a Thermo Scientific Orion 5 Star pH meter. Following the instructions in ISO 2917:1999, 5 g of each sample were collected, and the pH was then determined from the sample at a temperature of 20 ± 2 °C.

### 2.4. Statistical Analysis

Linear regression was used to study bacterial proliferation’s effect on gaseous production. Aerobic bacteria, LAB, and *Pseudomonas* spp. are the independent variables that can influence the production of gases, which were used as dependent variables in the analysis. Since it is essential to test the normality assumption when undertaking parametric statistical analyses, a Shapiro–Wilk test was used to assess the normality of the residuals of the linear regression results. All the analyses were conducted using JASP software (JASP, Amsterdam, The Netherlands).

## 3. Results

### 3.1. Quality and Shelf Life Assessment

**Figure 4:**
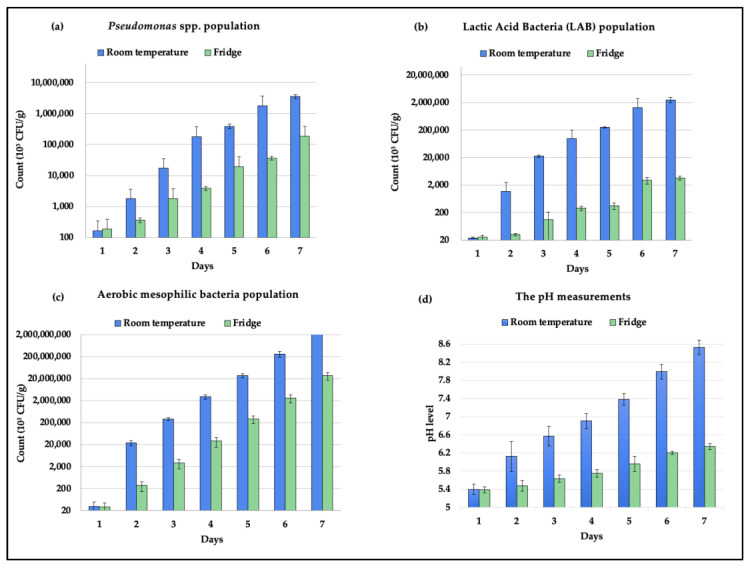
The population counts of (**a**) Pseudomonas, (**b**) LAB, (**c**) aerobic mesophilic bacteria, and (**d**) the pH measurements of the beef samples.

The microbial population counts of *Pseudomonas* spp., LAB, and aerobic bacteria in the beef samples in addition to the pH measurements are shown in Figure 4. The GCC Standards Organization’s microbiological food safety criteria were used to identify when the meat samples had reached their expiration dates (GSO 1016:2015). According to the GSO standards, the aerobic bacteria population count is the primary indicator for the expiration of beef, and a sample is considered spoiled when it exceeds the threshold of 10^6^ log CFU/g. Salmonella is another sign of beef’s expiration; a single colony is sufficient to deem a beef sample expired. Additionally, several researchers identified the count of various microbes, such as *Pseudomonades* spp. and lactic acid bacteria, etc., that indicate the spoilage occurrence. For instance, Odeyemi et al. indicated that raw meat samples expired when the *Pseudomonas* spp. and LAB population counts reached 10^7^–10^8^ CFU/g and 10^8^ CFU/cm^2^, respectively [38]. Therefore, the beef samples stored at room temperature expired on day 2 while the samples stored in the fridge expired on day 4, as illustrated in Figure 4c. However, the microbial population counting and the e-nose data collection were continued until day 7 to visualize the connection between the gas emissions and the microbial growth of *Pseudomonas* spp., LAB, and aerobic mesophilic bacteria.

### 3.2. Electronic Nose System

**Figure 5:**
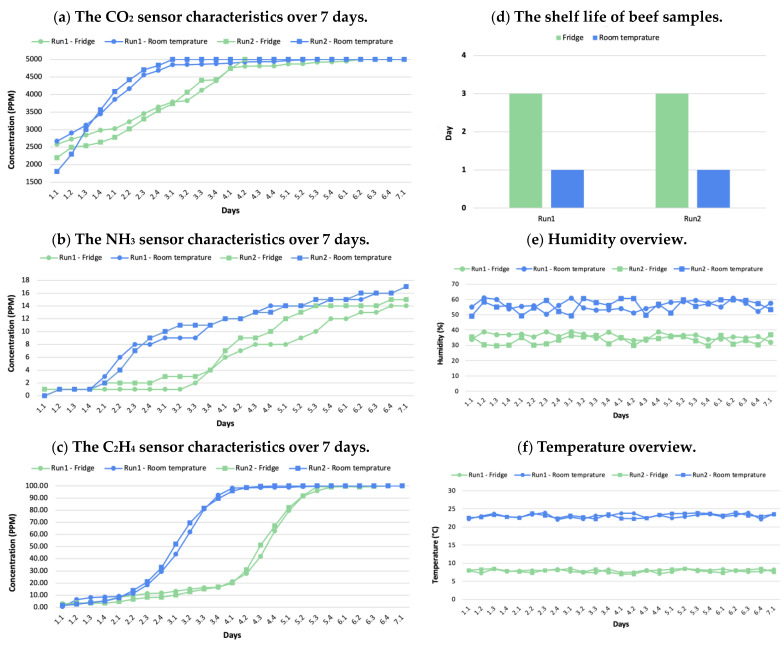
The electronic nose system data over the 7 day experimental period show (**a**) carbon dioxide gas emissions, (**b**) ammonia gas emissions, (**c**) ethylene gas emissions, (**d**) shelf life of the beef samples in different storage temperatures, (**e**) humidity overview, and (**f**) storage temperature overview.

Figure 5 presents the data collected from the e-nose system over a period of 7 days for two experimental runs for each storage temperature. As indicated via the microbial population counts in Section 3.1, the beef samples stored at room temperature and in the fridge expired on the second and fourth days, respectively. Hence, the shelf life of beef at room temperature is 1 day and the beef stored in the fridge is 3 days, as shown in Figure 5d. Therefore, the gas concentrations that correspond to beef spoilage were identified using the e-nose data on day 2 for the samples stored at room temperature and on day 4 for the samples stored in the fridge. Accordingly, the gas concentrations that correspond to the spoilage of beef range from 4552–4751 ppm for the CO_2_ gas sensor, 6–8 ppm for the NH_3_ gas sensor, and 18.4–21.1 ppm for the C_2_H_4_ gas sensor, as shown in Figure 5a–c.

### 3.3. Statistical Analysis

**Figure 6:**
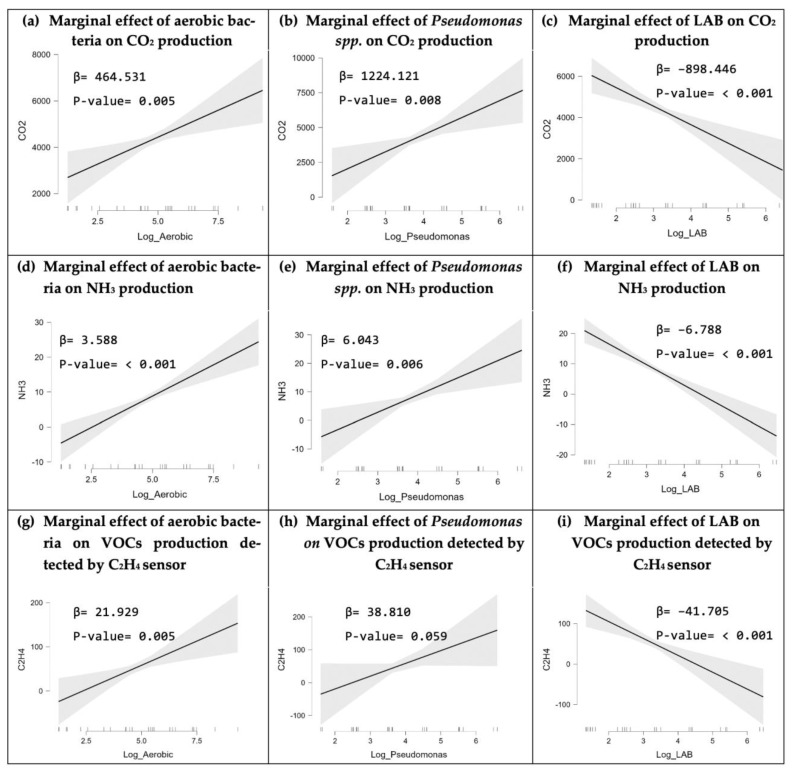
The linear regression results show the effect of various aerobic bacteria, *Pseudomonas* spp., and LAB on the VOCs detected by carbon dioxide, ammonia, and ethylene gas sensors.

The statistical analysis aimed to determine the impact of aerobic bacteria, LAB, and *Pseudomonas* spp. in beef on the production of carbon dioxide, ammonia, and ethylene. The unprocessed data of the bacterial proliferation and gas production were subjected to a normality test using the Shapiro–Wilk test. The *p*-value of the Shapiro–Wilk test results were less than the significance threshold of 5% for all the raw data, indicating that the residuals of the bacterial growth on the gas production are not normally distributed, which may introduce bias in the research results. To eliminate anomalous residual conditions, a Log transformation was applied to the microbial population data due to its high variance. After data transformation, the *p*-values of the Shapiro–Wilk test results were greater than 5%, which confirms that the linear regression residuals of aerobic bacteria, *Pseudomonas* spp., and LAB on carbon dioxide, ammonia, and ethylene production are normally distributed. Then, the regression analysis was carried out by looking at the marginal effect, regression coefficient (β), and *p*-value. The marginal effect visually demonstrates the effect of the independent variable (bacteria) on the dependent variable (gas). The regression coefficient (β) shows the amount of influence exerted by each bacterium on the gas production, while the *p*-value is used to determine whether the bacterial growth has a significant effect on the production of each gas. Figure 6 shows the marginal effect, regression coefficient (β), and *p*-value of each type of bacteria on the gas production.

#### 3.3.1. Linear Regression Result of Bacterial Growth on CO_2_ Production

The linear regression analysis depicted in Figure 6a shows a positive trend for the marginal effect of aerobic bacteria with a regression coefficient of 464.531. The *p*-value (0.005) is less than 5%, indicating a significant effect of aerobic bacteria on CO_2_ production. The analysis results show that the presence of more aerobic bacteria in beef will increase CO_2_ production, and vice versa. Likewise, Figure 6b shows that *Pseudomonas* spp. has a significant effect on CO_2_ production, as indicated by the *p*-value (0.008), which is less than the 5% significance level. The *Pseudomonas* spp. regression coefficient (1224.121) and the positive tendency in the marginal effect indicate that the presence of *Pseudomonas* spp. in beef increases CO_2_ production. In contrast to aerobic bacteria and *Pseudomonas* spp., Figure 6c presents the marginal effect of LAB, which demonstrates a negative trend as indicated by the regression coefficient of −898.446. The marginal effect, regression coefficient, and *p*-value are all less than 0.001, indicating that LAB has a statistically significant negative influence on CO_2_ production. Hence, the greater the concentration of LAB, the lower the CO_2_ production, and vice versa.

#### 3.3.2. Linear Regression Result of Bacterial Growth on NH_3_ Production

The linear regression analysis results in Figure 6d shows that the marginal effect of aerobic bacteria has a positive trend with a positive regression coefficient of 3.588 and a *p*-value of less than 0.001, which indicates the significant positive impact exerted by aerobic bacteria on the NH_3_ production. Hence, the rise in the aerobic bacteria population in beef will result in a higher NH_3_ production, and vice versa. Similarly, *Pseudomonas* spp. has a significant impact on NH3 production as shown in Figure 6e. The regression coefficient of *Pseudomonas* spp. (6.043) and the positive trend in the marginal effect indicates that the increase in the *Pseudomonas* spp. populations in beef triggers a greater NH_3_ production. Conversely, the marginal effect of LAB tends to show a negative trend, which is reinforced by the regression coefficient of −6.788 as shown in Figure 6f. The marginal effect, regression coefficient, and *p*-value < 0.001 indicate that LAB has a negative and significant impact on NH_3_ production. The greater the amount of LAB, the smaller the NH3 produced, and vice versa.

#### 3.3.3. Linear Regression Result of Bacterial Growth on VOCs Detected by C_2_H_4_ Sensor

The linear regression analysis depicted in Figure 6g reveals the positive impact of aerobic bacteria on the production of C_2_H_4_ or the VOCs that can be detected by the C_2_H_4_ sensor, particularly H_2_. The *p*-value (0.005), which is less than 5%, indicates a significant effect of aerobic bacteria on C_2_H_4_ production, and that the rise in aerobic bacteria in beef will increase the VOCs concentration. Nevertheless, *Pseudomonas* spp. was not proven to have a significant effect on gaseous production as indicated by a *p*-value greater than 5%, as shown in Figure 6h. In contrast, the marginal effect of LAB tends to demonstrate a negative trend, as indicated by the regression coefficient of −41.705 in Figure 6i. The marginal effect, regression coefficient, and *p*-value are all less than 0.001, indicating that LAB has a statistically significant negative influence on the production of C_2_H_4_ and/or H_2_.

## 4. Discussion

**Figure 7:**
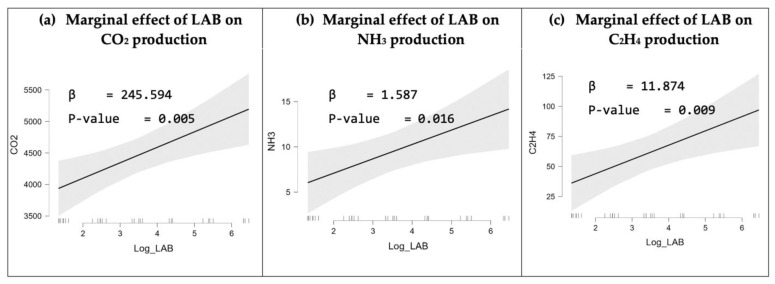
The linear regression results show the single effect of LAB on the production of carbon dioxide, ammonia, and ethylene.

Perishable quality monitoring during storage and transport is vital for the food industry as it helps in maintaining quality standards, reducing losses and waste, and ensuring that products are safe for human consumption. Conventional culturing methods are highly reliable and can accurately detect microbial spoilage based on the number of bacterial colonies. However, it is a very time-consuming and laborious method as the detection process takes 5–7 days and requires professional manpower, sterilized workspaces, sophisticated machines, and tools [39,40,41]. To overcome these drawbacks, numerous businesses in the food industry are currently investing in advanced technologies to monitor food quality and safety and many researchers are presenting various e-nose-based food monitoring systems that are highly accurate. However, most of the e-nose systems were designed to monitor the gases produced by either a specific type of food (i.e., chicken or meat) or by a limited selection of perishables, such as climacteric fruits. For instance, J. Brezmes et al. [42] and L. Ma et al. [43] highlighted e-nose systems that mainly monitor ethylene gas production to evaluate the ripeness levels and shelf life of a variety of fruits (i.e., apples, peaches, nectarines, pears, and kiwifruit), M. A. Putra [44] developed an e-nose system to assess the quality of milk by monitoring the NH_3_ and H_2_S levels, and Rivai et al. [29] evaluated meat freshness by monitoring the H_2_S and NH_3_ production [29,42,43,44]. Therefore, we developed a cost-effective, noninvasive, and versatile food quality monitoring system that can evaluate the freshness of a variety of food items, including meats, fruits, and vegetables. This was enabled by our gas sensor selection, which can monitor the major VOCs produced from meats and fresh produce. A carbon dioxide gas sensor was selected as CO_2_ is a primary by-product of bacterial activities [24,30]. An ammonia sensor was also selected as NH_3_ is an important signal of the deterioration of meats and protein-rich foods [31,32]. Similarly, an ethylene sensor was chosen because it is a primary indicator of the maturity of numerous fresh produce items, so monitoring its concentration is important for controlling the ripening process [24,33]. Moreover, the ME3-C2H4 ethylene sensor that was used in this research can also respond to a few other gases, primarily hydrogen [45]. In addition, humidity and temperature sensors were included to assess the environmental conditions during storage and transportation as their fluctuation can significantly influence the shelf life and overall food quality. Hence, the sensor selection causes our e-nose system to be applicable to various fresh food industries. In this research, we used the IoT-enabled e-nose system to monitor the VOCs emissions of raw beef to identify the levels that correspond to spoilage by correlating the e-nose readings with the conventional plate count results. When beef undergoes spoilage, the biochemical and physiochemical activities result in the production of amino acids, causing the meat to be vulnerable to microbial activities and growth [46]. As a result, volatile compounds such as CO_2_, NH_3_, and H_2_S are produced. Therefore, the concentration of nitrogen and hydrogen compounds can be used for evaluating the freshness of meat in the market as well as during storage [47,48]. Various microorganisms populate the beef substrate and produce a variety of gases that can be used to predict spoilage proximity. However, the impact of different bacteria that populate the beef substrate (i.e., *Pseudomonas* spp., LAB, and aerobic bacteria) on the production of VOCs has not been thoroughly investigated. Several studies associated LAB with the production of CO_2_, H_2_, and NH_3_ during the spoilage of beef [49,50]. However, the gaseous contribution of LAB in comparison to the contributions of other microorganisms in beef has not been evaluated by these studies. Hence, evaluating the gaseous production of these bacteria while they are in the same medium to comprehend the indications of the emissions of VOCs is important. For instance, although the LAB proliferation was significant in the beef samples, as shown in Figure 4b, their contribution to the production of gases was minimal, as illustrated in Figure 6c,f,i. This could be due to the dominance of facultatively heterofermentative LAB species in the beef samples, which are characterized by producing two moles of lactate from glucose without gas formation [50,51]. To validate that, the singular effect of LAB bacteria on gas production was analyzed without including the effect of *Pseudomonas* spp. nor aerobic bacteria in the regression model. The test results in Figure 7 show a positive influence of LAB on all VOC production. This contradicts the findings in Figure 6c,f,i, which demonstrates that the interactions between LAB bacteria and other bacteria in beef might lessen the gas generation engendered by LAB bacteria.

Thus, it can be concluded that the production of VOC concentrations in beef could be used to indicate the proliferation of aerobic bacteria and pseudomonas. Although different volatile compounds are produced by different bacteria, CO_2_ was identified by performing the headspace technique as a predominant gas that is produced by various species in beef [49]. In addition, the amines are among the metabolites generated by the activity of *Pseudomonas* spp. utilizing nitrogenous substances in raw beef [50,52]. This explains the positive marginal effect trends of *Pseudomonas* spp. and aerobic bacteria on the production of VOCs (Figure 7). The e-nose system demonstrated that the gas concentrations recorded by the carbon dioxide, ammonia, and ethylene sensors at the time the beef expired ranged from 552 ppm to 4751 ppm, 6 ppm to 8 ppm, and 18.4 ppm to 21.1 ppm, respectively. After the spoilage occurrence, the CO_2_ concentration and the concentrations of the gases detected by the ethylene sensor, predominantly C_2_H_4_ and H_2_, increased rapidly. This results in the saturation of the carbon dioxide and ethylene sensors as they reached their maximum detection ranges of 100 ppm and 5000 ppm, respectively, indicating that there are no more active sites available to react with new gas molecules as the concentrations rise. The identified gas concentration ranges that correspond to the beef spoilage can be utilized as a baseline to detect beef deterioration in the future using only the electronic nose, thus removing the need of using conventional microbiological plate counts. However, a data set that contains the gas concentrations associated with the shelf lives of various food items (e.g., fruits, vegetables, seafood, etc.) is required for the effective utilization of the electronic nose in smart food systems. The shelf-life estimation of different food items is a pivotal piece of information that will define this system’s intelligence. For that, our future research would extend to include more gas sensors in the e-nose system, identify the gas concentration levels produced from a wider range of fresh foods (i.e., meats and fresh produce), and develop a prediction model for spoilage and ripening classification. In addition, since glucose consumption is of crucial significance for bacterial growth and their by-products, our future research may extend to include the glucose content in raw beef in the linear regression analysis of bacterial proliferation and VOC production.

## 5. Conclusions

This paper presents an IoT-enabled e-nose system for food quality monitoring. Raw beef was used to evaluate the system’s performance. The VOC concentrations produced from the beef samples stored at 4 °C and the two stored at 21 °C were evaluated using the e-nose system that utilizes a carbon dioxide gas sensor, ammonia gas sensors, and ethylene gas sensors. Microbial population quantifications of aerobic bacteria, *Pseudomonas* spp., and LAB were conducted to evaluate beef quality and identify spoilage. The production of VOCs was correlated with the proliferation of bacteria using linear regression, and it was discovered that aerobic bacteria and *Pseudomonas* spp. play a significant role in the production of VOCs in raw beef, as opposed to LAB. The VOC concentrations that are associated with raw beef spoilage were identified using the e-nose system and can be used in the future to detect beef spoilage without needing to use microbial plate counts.

## Figures and Tables

**Table 1:** Sensors’ specifications.

Sensor	Detection Range	Accuracy	Response Time	Operating Temperatures
ZE03-NH3	1–100 ppm	±1 ppm	≤150 s	From 0 °C to 50 °C
ZE03-C2H4	0–100 ppm	±0.1 ppm	≤30 s	From 0 °C to 50 °C
MH-Z19C	400–5000 ppm	±1 ppm	≤120 s	From −10 °C to 50 °C
AM2302	From −40 °C to 80 °C and 0–100% RH	±0.5 °C and ±0.3% RH	≤5 s	From −40 °C to 80 °C

## Data Availability

The data presented in this study are available on request from the corresponding author.

## References

[1] U. N. E. Programme (2021). Food Waste Index Report..

[2] Kustar A., Patino-Echeverri D. (2021). A review of environmental life cycle assessments of diets: Plant-based solutions are truly sustainable, even in the form of fast foods. Sustainability.

[3] Costantini M., Vazquez-Rowe I., Manzardo A., Bacenetti J. (2021). Environmental impact assessment of beef cattle production in semi-intensive systems in Paraguay. Sustain. Prod. Consump..

[4] Damdam A.N., Alzahrani A., Salah L., Salama K.N. (2023). Effects of UV-C Irradiation and Vacuum Sealing on the Shelf-Life of Beef, Chicken and Salmon Fillets. Foods.

[5] Ishangulyyev R., Kim S., Lee S.H. (2019). Understanding food loss and waste—Why are we losing and wasting food?. Foods.

[6] Stenmarck Â., Jensen C., Quested T., Moates G., Buksti M., Cseh B., Juul S., Parry A., Politano A., Redlingshofer B. (2016). Estimates of European Food Waste Levels.

[7] Wickramasinghe N.N., Ravensdale J., Coorey R., Chandry S.P., Dykes G.A. (2019). The predominance of psychrotrophic pseudomonads on aerobically stored chilled red meat. Compr. Rev. Food Sci. Food Saf..

[8] Yim D.G., Jin S.K., Hur S.J. (2019). Microbial changes under packaging conditions during transport and comparison between sampling methods of beef. J. Anim. Sci. Technol..

[9] Wang J., Wang H., He J., Li L., Shen M., Tan X., Min H., Zheng L. (2015). Wireless sensor network for real-time perishable food supply chain management. Comput. Electron. Agric..

[10] Prabhakar P.K., Vatsa S., Srivastav P.P., Pathak S.S. (2020). A comprehensive review on freshness of fish and assessment: Analytical methods and recent innovations. Food Res. Int..

[11] Ajaykumar V., Mandal P.K. (2020). Modern concept and detection of spoilage in meat and meat products. Meat Quality Analysis.

[12] Fletcher B., Mullane K., Platts P., Todd E., Power A., Roberts J., Chapman J., Cozzolino D., Chandra S. (2018). Advances in meat spoilage detection: A short focus on rapid methods and technologies. CyTA-J. Food.

[13] Lohumi S., Lee S., Lee H., Cho B.-K. (2015). A review of vibrational spectroscopic techniques for the detection of food authenticity and adulteration. Trends Food Sci. Technol..

[14] Yoon J., Lee K., Park Y. (2016). A simple and rapid method for detecting living microorganisms in food using laser speckle decorrelation. arXiv.

[15] Jung Y., Min J., Choi J., Bang J., Jeong S., Pyun K.R., Ahn J., Cho Y., Hong S., Hong S. (2022). Smart paper electronics by laser-induced graphene for biodegradable real-time food spoilage monitoring. Appl. Mater. Today.

[16] Dong D., Jiao L., Li C., Zhao C. (2019). Rapid and real-time analysis of volatile compounds released from food using infrared and laser spectroscopy. TrAC Trends Anal. Chem..

[17] Poghossian A., Geissler H., Schöning M.J. (2019). Rapid methods and sensors for milk quality monitoring and spoilage detection. Biosens. Bioelectron..

[18] Nowshad F., Khan M.S. (2021). Electronic tongue for food safety and quality assessment. Techniques to Measure Food Safety and Quality.

[19] Kaur G., Bhari R., Kumar K. (2022). Electronic noses and tongue-based sensor systems in food science. Nanosensing and Bioanalytical Technologies in Food Quality Control.

[20] Shi J., Xiao Y., Jia C., Zhang H., Gan Z., Li X., Yang M., Yin Y., Zhang G., Hao J. (2022). Physiological and biochemical changes during fruit maturation and ripening in highbush blueberry (*Vaccinium corymbosum*). Food Chem..

[21] Jiang S., Liu Y. (2020). Gas sensors for volatile compounds analysis in muscle foods: A review. TrAC Trends Anal. Chem..

[22] Thakur M., Ragavan K. (2013). Biosensors in food processing. J. Food Sci. Technol..

[23] Loudiyi M., Temiz H.T., Sahar A., Haseeb Ahmad M., Boukria O., Hassoun A., Aït-Kaddour A. (2022). Spectroscopic techniques for monitoring changes in the quality of milk and other dairy products during processing and storage. Crit. Rev. Food Sci. Nutr..

[24] Shaalan N.M., Ahmed F., Saber O., Kumar S. (2022). Gases in food production and monitoring: Recent advances in target chemiresistive gas sensors. Chemosensors.

[25] Ali M.M., Hashim N., Aziz S.A., Lasekan O. (2020). Principles and recent advances in electronic nose for quality inspection of agricultural and food products. Trends Food Sci. Technol..

[26] Wang M., Gao F., Wu Q., Zhang J., Xue Y., Wan H., Wang P. (2018). Real-time assessment of food freshness in refrigerators based on a miniaturized electronic nose. Anal. Methods.

[27] Sanaeifar A., Mohtasebi S.S., Ghasemi-Varnamkhasti M., Ahmadi H., Lozano J. (2014). Development and application of a new low cost electronic nose for the ripeness monitoring of banana using computational techniques (PCA, LDA, SIMCA, and SVM). Czech J. Food Sci..

[28] Rivai M., Budiman F., Purwanto D., Al Baid M.S.A., Tukadi, Aulia D. (2022). Discrimination of durian ripeness level using gas sensors and neural network. Procedia Comput. Sci..

[29] Rivai M., Budiman F., Purwanto D., Simamora J. (2018). Meat freshness identification system using gas sensor array and color sensor in conjunction with neural network pattern recognition. J. Theor. Appl. Inf. Technol..

[30] Weston M., Geng S., Chandrawati R. (2021). Food sensors: Challenges and opportunities. Adv. Mater. Technol..

[31] Matindoust S., Farzi G., Nejad M.B., Shahrokhabadi M.H. (2021). Polymer-based gas sensors to detect meat spoilage: A review. React. Funct. Polym..

[32] Zhang D., Yu S., Wang X., Huang J., Pan W., Zhang J., Meteku B.E., Zeng J. (2022). UV illumination-enhanced ultrasensitive ammonia gas sensor based on (001) TiO_2_/MXene heterostructure for food spoilage detection. J. Hazard. Mater..

[33] Matindoust S., Baghaei-Nejad M., Abadi M.H.S., Zou Z., Zheng L.-R. (2016). Food quality and safety monitoring using gas sensor array in intelligent packaging. Sens. Rev..

[34] Falagan N., Terry L.A. (2018). Recent advances in controlled and modified atmosphere of fresh produce. Johns. Matthey Technol. Rev..

[35] da Costa T.P., Gillespie J., Cama-Moncunill X., Ward S., Condell J., Ramanathan R., Murphy F. (2023). A systematic review of real-time monitoring technologies and its potential application to reduce food loss and waste: Key elements of food supply chains and IoT technologies. Sustainability.

[36] Reese K.L., Rasley A., Avila J.R., Jones A.D., Frank M. (2020). Metabolic profiling of volatile organic compounds (VOCs) emitted by the pathogens *Francisella tularensis* and *Bacillus anthracis* in liquid culture. Sci. Rep..

[37] Chen J., Tang J., Shi H., Tang C., Zhang R. (2017). Characteristics of volatile organic compounds produced from five pathogenic bacteria by headspace-solid phase micro-extraction/gas chromatography-mass spectrometry. J. Basic Microb..

[38] Odeyemi O.A., Alegbeleye O.O., Strateva M., Stratev D. (2020). Understanding spoilage microbial community and spoilage mechanisms in foods of animal origin. Compr. Rev. Food Sci. Food Saf..

[39] Ferone M., Gowen A., Fanning S., Scannell A.G.M. (2020). Microbial detection and identification methods: Bench top assays to omics approaches. Compr. Rev. Food Sci. Food Saf..

[40] Böhme K., Cremonesi P., Severgnini M., Villa T.G., Fernández-No I.C., Velázquez J.B., Castiglioni B., Calo-Mata P. (2014). Detection of food spoilage and pathogenic bacteria based on ligation detection reaction coupled to flow-through hybridization on membranes. Biomed. Res. Int..

[41] Paniel N., Noguer T. (2019). Detection of salmonella in food matrices, from conventional methods to recent aptamer-sensing technologies. Foods.

[42] Brezmes J., Fructuoso M., Llobet E., Vilanova X., Recasens I., Orts J., Saiz G., Correig X. (2005). Evaluation of an electronic nose to assess fruit ripeness. IEEE Sens. J..

[43] Ma L., Wang L., Chen R., Chang K., Wang S., Hu X., Sun X., Lu Z., Sun H., Guo Q. (2016). A low cost compact measurement system constructed using a smart electrochemical sensor for the real-time discrimination of fruit ripening. Sensors.

[44] Putra M.A., Rivai M., Arifin A. (2018). Milk assessment using potentiometric and gas sensors in conjunction with neural network. Proceedings of the 2018 International Seminar on Intelligent Technology and Its Applications (ISITIA).

[45] Winsen Co. Electrochemical Ethylene Gas Sensor ME3-C2H4. 9 July 2021. https://www.winsen-sensor.com/d/files/me3-c2h4-0-100ppm(ver1_1)-manual.pdf.

[46] Nastiti P., Bintoro N., Karyadi J., Rahayoe S., Nugroho D. (2022). Classification of freshness levels and prediction of changes in evolution of NH3 and H2S gases from chicken meat during storage at room temperature. J. Tek. Pertan. Lampung J. Agric. Eng..

[47] Pavase T.R., Lin H., Shaikh Q.-U.-A., Hussain S., Li Z., Ahmed I., Lv L., Sun L., Shah S.B.H., Kalhoro M.T. (2018). Recent advances of conjugated polymer (CP) nanocomposite-based chemical sensors and their applications in food spoilage detection: A comprehensive review. Sens. Actuators B Chem..

[48] Edita R., Darius G., Vinauskienė R., Eisinaitė V., Balčiūnas G., Dobilienė J., Tamkutė L. (2018). Rapid evaluation of fresh chicken meat quality by electronic nose. Czech J. Food Sci..

[49] Hernández-Macedo M., Contreras-Castillo C., Tsai S., Da Cruz S., Sarantopoulos C., Padula M., Dias C. (2012). Gases and volatile compounds associated with micro-organisms in blown pack spoilage of Brazilian vacuum-packed beef. Lett. Appl. Microbiol..

[50] Casaburi A., Piombino P., Nychas G.-J., Villani F., Ercolini D. (2015). Bacterial populations and the volatilome associated to meat spoilage. Food Microbiol..

[51] Kandler O. (1983). Carbohydrate metabolism in lactic acid bacteria. Antonie Van Leeuwenhoek.

[52] Met A., Yesilcubuk N.S. (2017). Comparison of two volatile sampling techniques based on different loading factors in determination of volatile organic compounds released from spoiled raw beef. Food Anal. Methods.

